# Advanced strategies to enhance the safety, persistence, and efficacy of CAR-T cells in solid tumors

**DOI:** 10.3389/fimmu.2026.1813730

**Published:** 2026-05-20

**Authors:** Iqra Ajmal, Bingtan Du, Na Huang, Qianying Huang, Dan Jiang, Muhammad Asad Farooq, Guangxian Xu

**Affiliations:** 1 Guangdong Provincial Key Laboratory of Medical Immunology and Molecular Diagnostics, The First Dongguan Affiliated Hospital, School of Medical Technology, Guangdong Medical University, Dongguan, Guangdong, China; 2 Shanghai Key Laboratory of Regulatory Biology, School of Life Sciences, East China Normal University, Shanghai, China

**Keywords:** CAR-T cell therapy, chemokine trafficking, chimeric antigen receptor-T cells, cytokine release syndrome, genome engineering, immune checkpoint regulation, immunotherapy, tumor microenvironment

## Abstract

Chimeric antigen receptor (CAR) T-cell therapy has revolutionized the treatment of hematologic cancers but encounters challenges, including severe treatment-related toxicities, a highly suppressive tumor microenvironment (TME), limited long-term persistence, and poor trafficking/infiltration into solid tumors. This review outlines recent genetic engineering strategies to address these issues and enhance the safety, durability, and efficacy of CAR-T cell therapy. To reduce cytokine release syndrome and neurotoxicity, methods such as affinity-tuned and humanized scFvs, hinge/TM optimization, and ITAM calibration have been developed, along with programmable “switch-off” and “switch-on” systems that include suicide genes, antibody-bridging switches, and optogenetic or hypoxia-gated circuits. TME remodeling strategies utilize nanomaterials for targeted cytokine delivery, cell-surface “backpack” systems, and engineered oncolytic viruses that release cytokines or checkpoint-blocking agents. For durability and resistance to exhaustion, precise genome engineering techniques, including CRISPR-based editing and multiplexed shRNA platforms, were employed to target inhibitory receptors and exhaustion-driving transcriptional programs. Additionally, chemokine-receptor engineering and local biomaterial-based delivery systems are discussed as ways to enhance CAR-T trafficking and intratumoral persistence. These innovations collectively point toward integrated, patient-specific CAR-T platforms that incorporate safety controls, metabolic and transcriptional flexibility, and enhanced trafficking through the TME to broaden clinical use.

## 1 Introduction

Chimeric antigen receptor (CAR) T-cell therapy represents a groundbreaking advancement in immunotherapy, offering a highly personalized approach to the treatment of certain hematologic malignancies. CAR-T cells are genetically engineered T lymphocytes designed to recognize and eliminate the target cancer cells. This is achieved by modifying a patient’s autologous T cells to express synthetic receptors, known as CARs, which target specific tumor-associated antigens (TAAs). Unlike endogenous T cell receptors, CARs recognize antigens independently of major histocompatibility complex (MHC) presentation, enabling direct and potent immune responses against malignant cells (1). The clinical success of this strategy is underscored by multiple U.S. Food and Drug Administration (FDA)-approved CAR-T cell products, which have demonstrated remarkable remission rates in patients with relapsed or refractory cancers, including leukemia, lymphoma, and multiple myeloma (2–5). These milestones validate both the safety and therapeutic efficacy of CAR-T cell therapy and have accelerated further research and development in this rapidly evolving field.

**Figure 1:**
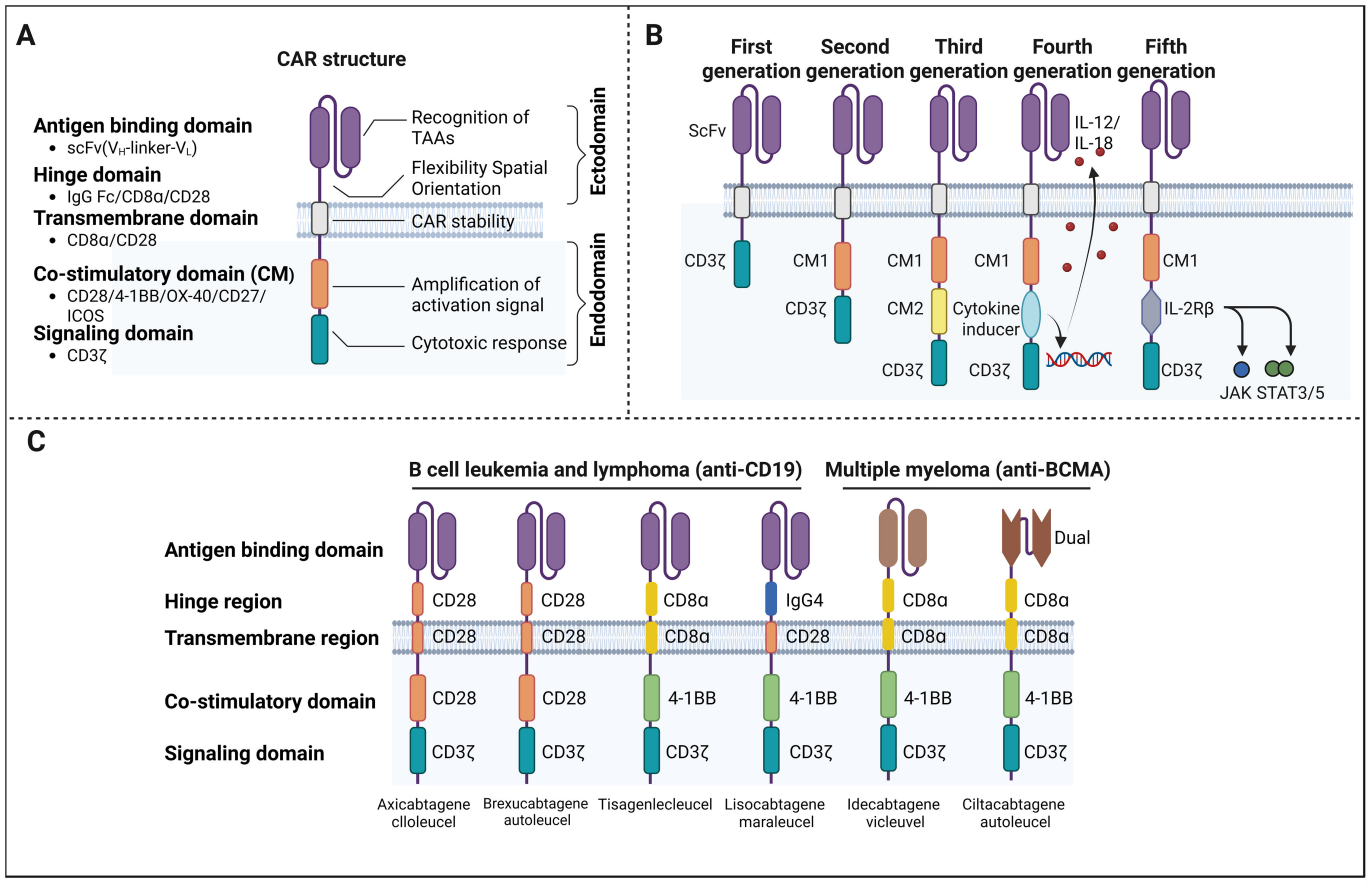
Structure, evolution, and clinical translation of CAR-T cell therapy. **(A)** Schematic overview of the modular CAR structure, including the antigen-binding scFv, hinge/spacer, transmembrane domain, and intracellular signaling modules. **(B)** Progressive evolution of CAR designs from first- to fifth-generation constructs, reflecting increasing signaling complexity and functional refinement. **(C)** Overview of FDA-approved CAR-T cell therapies and their primary indications in hematologic malignancies.

The CAR molecule comprises several integrated domains that collectively enable T cells to recognize and eliminate tumor cells (6). At the extracellular level, a single-chain variable fragment (scFv) derived from an antibody confers antigen specificity by directly binding to a TAA. This antigen-binding domain is linked to a hinge or spacer region, which provides structural flexibility and optimal spatial orientation for target engagement (7). The transmembrane domain anchors the CAR into the T cell membrane and contributes to receptor stability. Intracellularly, the CAR contains a signaling domain (ICD), typically derived from CD3ζ, and, in more advanced designs, one or more co-stimulatory domains that enhance T cell activation, proliferation, and survival (8) (**Figure 1A**). The modular nature of CAR architecture has enabled systemic optimization, driving the evolution of successive CAR generations with progressively enhanced functionality.

First-generation CARs were the most basic, consisting solely of a CD3ζ signaling domain. Although they could initiate T cell activation upon antigen recognition, the absence of co-stimulatory signaling led to poor T cell proliferation, limited persistence, and ultimately weak clinical responses, particularly in solid tumors (9, 10). Second-generation CARs addressed these limitations by incorporating a single co-stimulatory domain, typically CD28 or 4-1BB (CD137), in addition to CD3ζ. This modification significantly improved CAR-T cell expansion, cytokine secretion, and *in vivo* persistence, leading to more robust and durable antitumor responses (11) (**Figure 1B**). Clinically, second-generation CARs form the backbone of most FDA-approved CAR-T cell therapies and have achieved significant success in B-cell malignancies (**Figure 1C**) (1, 12).

Third-generation CARs further expanded upon this concept by integrating two co-stimulatory domains to amplify the intracellular signaling. While preclinical studies suggested enhanced antitumor potency and resistance to T cell exhaustion, clinical trials have yielded mixed results, with no consistent evidence of superiority over second-generation CARs to date (13–15). Fourth-generation CARs, also known as T cells redirected for universal cytokine-mediated killing (TRUCKs), introduced an additional functional layer by incorporating genes encoding pro-inflammatory cytokines, such as interleukin-12 (IL-12). Beyond direct tumor cell killing, these CAR-T cells actively remodel the tumor microenvironment (TME) by recruiting and activating innate immune components. Although this strategy holds promise for overcoming immunosuppression in solid tumors, the risk of excessive inflammation and off-target toxicity remains a significant concern. More recently, fifth-generation CARs have been developed to integrate cytokine receptor signaling domains, thereby more closely mimicking physiological T cell activation pathways and further enhancing antitumor efficacy (11, 16, 17). Despite these advances, the broader application of CAR-T cell therapy, particularly in solid tumors, remains limited by several critical challenges. These include severe treatment-related toxicities, inefficient trafficking and infiltration of CAR-T cells into tumor sites, the profoundly immunosuppressive nature of the TME, and insufficient long-term persistence of infused cells (18). Addressing these barriers has become a central focus of current research. Increasingly, interdisciplinary approaches drawing on synthetic biology, materials science, and systems immunology are being leveraged to enhance CAR-T cell safety, functionality, and durability. However, a significant tension exists between the increasing biological sophistication of these next-generation platforms and the practicalities of clinical manufacturing, regulatory approval, and economic sustainability. This review highlights recent innovations aimed at overcoming these key limitations and provides a critical appraisal of their translational maturity and the specific failure modes that govern their clinical implementation.

## 2 Engineering strategies for enhanced CAR-T safety

CAR-T cell therapy has revolutionized cancer treatment, yet its clinical applications are frequently limited by significant toxicities, which remain a major barrier to broader adoption, particularly in solid tumors. The most common complication is cytokine release syndrome (CRS), an acute systemic inflammatory response triggered by rapid activation and expansion of CAR-T cells, resulting in the massive release of cytokines such as IL-6, interferon-gamma (IFN-γ), and tumor necrosis factor-alpha (TNF-α) (19, 20). Severe CRS can progress to hypotension, multi-organ dysfunction, and even fatal outcomes if not promptly managed.

Neurological toxicities also pose substantial challenges to CAR-T cell therapy. Immune effector cell-associated neurotoxicity syndrome (ICANS) encompasses encephalopathy, seizures, and neuropsychiatric symptoms and is thought to arise from endothelial activation and cytokine-mediated blood-brain barrier disruption (19, 21, 22). The risk and severity of both CRS and ICANS are closely linked to CAR T-cell dose, proliferative capacity, and cytokine production, particularly in patients with high tumor burden (23). While structural modifications aim to mitigate these risks, the relationship between CAR architecture and CRS/ICANS remains highly complex and often construct-dependent. Adaptive strategies such as split-dose infusion and patient-specific dosing are increasingly employed to mitigate these risks (24, 25).

To improve CAR-T cell therapy, innovative strategies have emerged to mitigate toxicity while maintaining therapeutic potential. The self-regulating CARs, “switch-on/switch-off CARs,” have been developed by integrating active control mechanisms with external modulators, such as suicide genes and responsive sensors that detect light, sound, or oxygen levels, allowing clinicians to fine-tune CAR-T cell activity in real time. These strategies represent a spectrum of translational maturity, with suicide switches being clinically established while spatiotemporal control systems (e.g., optogenetics) remain in the early preclinical proof-of-concept stage (26). These mechanistic advances underscore the potential to overcome toxicity as a critical barrier, paving the way for safer, more effective CAR-T therapies. In the following sections, we will focus on structural modifications and emerging strategies to manage these toxicities and improve therapeutic outcomes.

### 2.1 Structural optimization and self-regulating CAR designs

One strategy to mitigate CAR-T cell toxicity is to fine-tune the CAR’s binding affinity for TAAs. By lowering affinity, CAR-T cells preferentially target tumor cells with high antigen expression while sparing healthy tissues with low antigen levels. Recent research suggests that altering the binding affinity of the extracellular domain of CARs profoundly affects their activation threshold, cytokine release, and toxicity profiles (27).

In a study by Ghorashian et al., a CD19-targeted CAR with reduced antigen-binding affinity, termed CAT, was developed in contrast to the conventional FMC63-based construct. Despite its lower affinity, the CAT-CAR-T cells demonstrated enhanced antitumor activity, achieving complete remission in more than 80% of treated pediatric patients (12 of 14) with replaced or refractory B-cell acute lymphoblastic leukemia (B-ALL). Importantly, this enhanced efficacy was accompanied by an improved safety profile, with no reported cases of severe CRS and only one instance of ICANS (28). These findings suggest that optimized CAR affinity can uncouple antitumor efficacy from excessive immune activation.

Humanizing CAR constructs is another approach that replaces murine-derived scFv regions with human counterparts, reducing immunogenicity, limiting immune-mediated rejection, and enhancing CAR-T cell persistence. A prominent clinical example of this transition is huCART19 (NCT02374333), which utilizes a humanized anti-CD19 scFv. While the original murine-derived FMC63 scFv is highly effective, it can trigger human anti-mouse antibody (HAMA) responses, leading to T-cell rejection. In Phase I trials, huCART19 demonstrated durable persistence and successfully induced remissions in patients who had previously relapsed after treatment with murine-based CAR-T cells, highlighting the clinical value of reducing immunogenicity to enhance long-term surveillance (29). Additionally, a humanized CAR containing the human-derived CD19-scFv with BAFFR single-variable domains showed excellent *in vitro* and *in vivo* efficacy. These CAR-T cells were less exhausted and exhibited superior tumor eradication (30).

Beyond antigen-binding domains, the hinge and transmembrane domain (HD/TMD) play a pivotal role in regulating CAR-T cell activation and toxicity. In specific clinical contexts, the integration of a humanized scFv with a CD8α HD/TMD has been associated with reduced treatment-related toxicities. However, the extent of this reduction may depend on the specific target antigen and tumor burden, suggesting that the protective effect of HD/TMD configuration is construct-dependent. For instance, Hu19-CD19ζ CAR-T cells exhibited substantially fewer ICANS cases (4%) than traditional FMC63-based CD19-CAR-T cells (50%). This enhanced safety was due to low cytokine levels in the blood of patients receiving humanized CAR-T cells, which were dependent on the CAR’s HD/TMD configuration (31–33). Variations in the type and length of the HD/TMD may also influence toxicity levels (34). CAR-T cells with longer CD8α-HD/TMD domains were associated with lower cytokine production and a lower incidence of severe CRS or ICANS among patients (n=25) with refractory B-cell lymphoma (35, 36).

**Figure 2:**
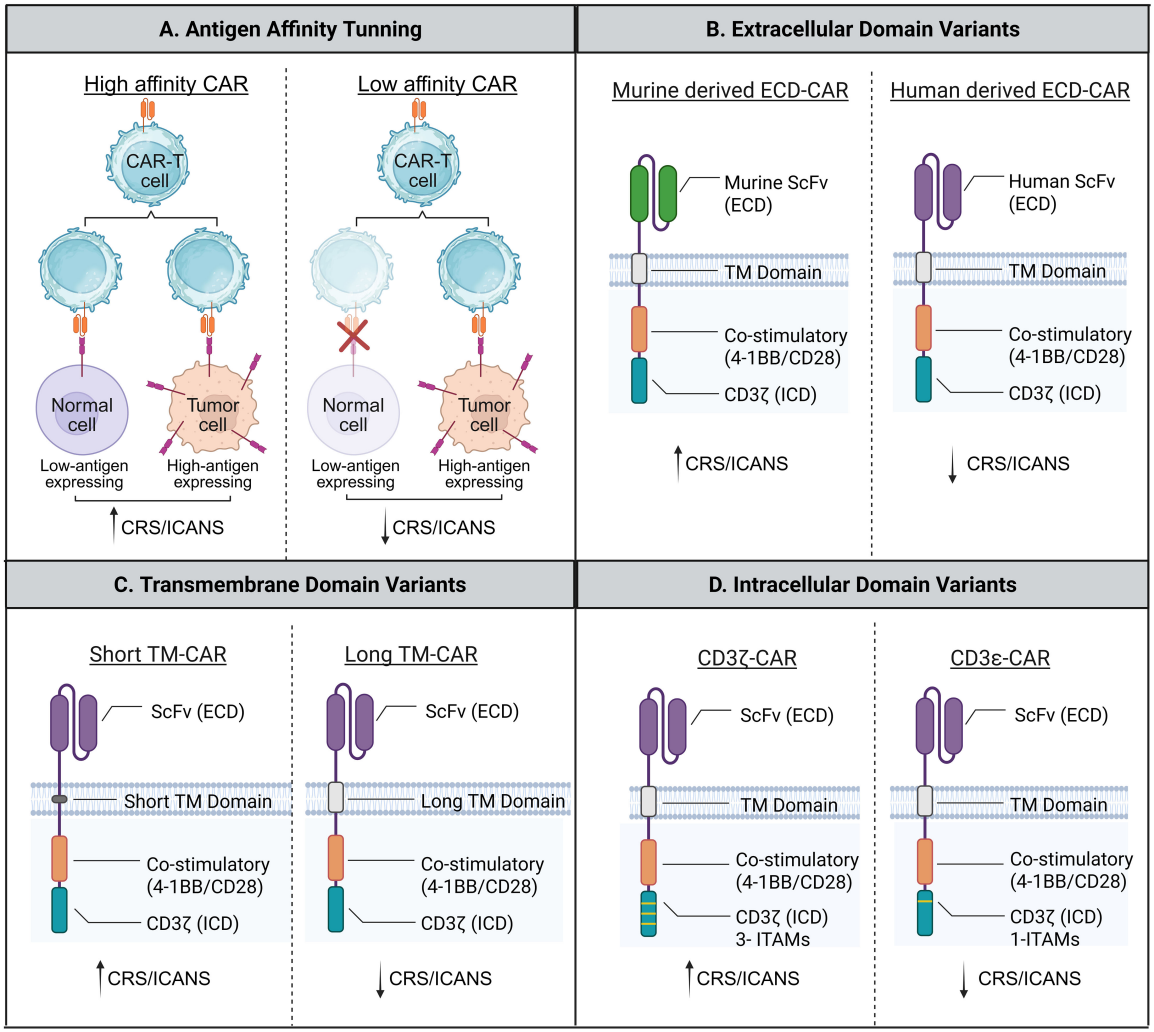
Structural strategies for self-regulating CAR-T cell activity. Schematic representations of CAR design features that modulate strength and toxicity. **(A)** Reduced antigen-binding affinity enhances discrimination between high-antigen-expressing tumor cells and low-antigen-expressing normal cells. **(B)** Humanized scFv reduces immunogenicity and supports CAR-T cell persistence. **(C)** Hing and transmembrane domain variants alter signaling intensity and cytokine output. **(D)** Modification of the CD3 signaling domain, including reduction in ITAM number, constrains excessive activation and cytokine release.

Intracellular signaling domains (ICD) also critically influence CAR-T cell function and toxicity. CARs incorporating the 4-1BB co-stimulatory domain generally induce more controlled and sustained activation compared with CD28-based CARs (37). This difference may be attributed to the THEMIS-SHP1 complex, which decreases CD3ζ phosphorylation and limits excessive cytokine secretion, including IFN-γ (38, 39). Furthermore, reducing the number of immunoreceptor Tyrosine-based activation motifs (ITAMs) within the CD3ζ domain, or substituting CD3ζ with alternative single-ITAM subunits, is hypothesized to limit CAR-T cell hyperactivation. While preclinical models suggest this reduces CRS risk, the functional implications for long-term antitumor surveillance require further validation across different malignancies (40). **Figure 2** illustrates these strategies for self-regulating CAR design.

### 2.2 The ‘switch off’ CARs

Switch-off CARs are designed to enable conditional termination of CAR-T cell activity via internal or externally administered triggers, providing an additional layer of safety. These systems are crucial for preventing the severe, systemic side effects associated with related toxicities such as CRS and ICANS (41).

**Figure 3:**
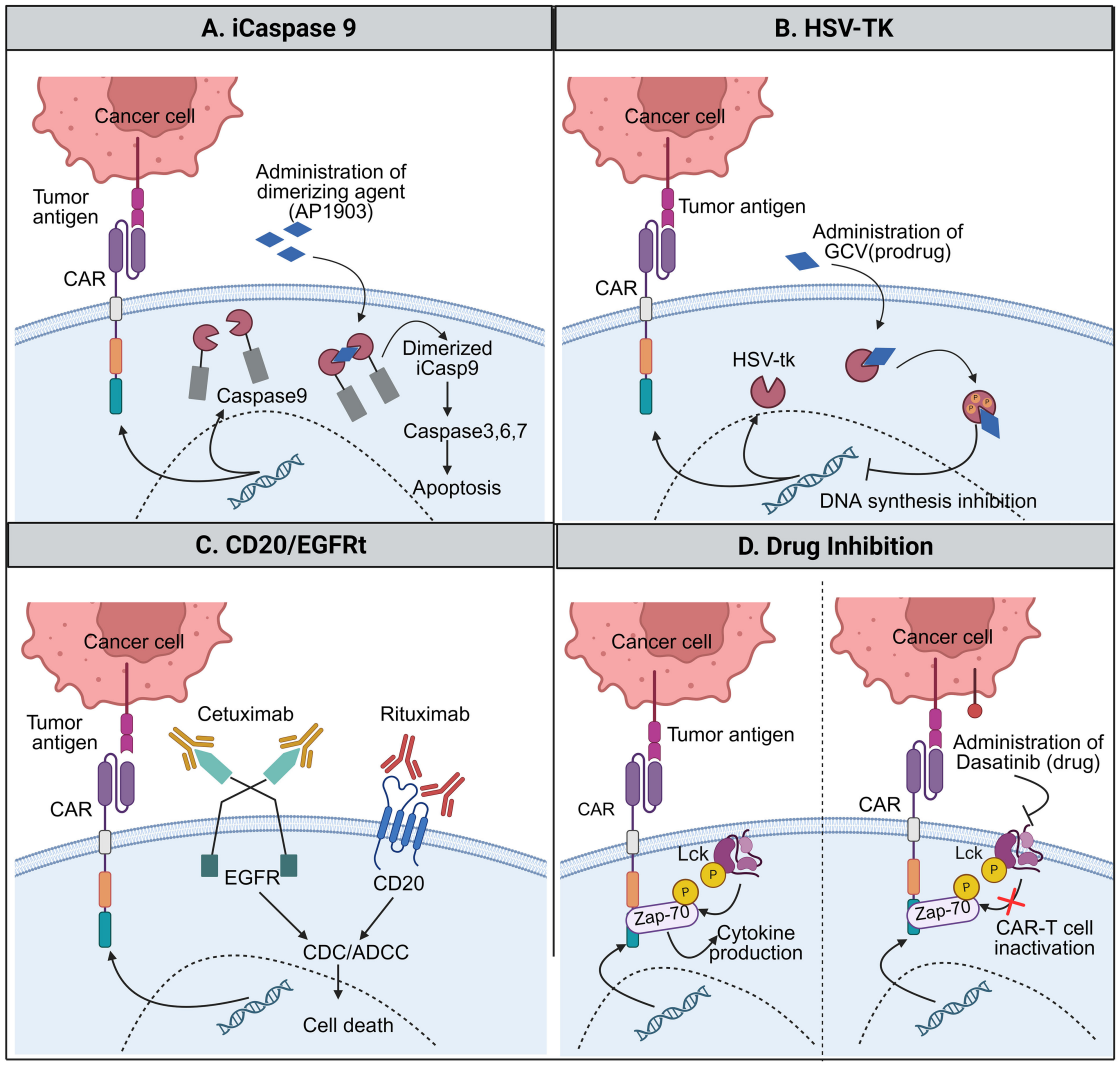
Switch-off CAR strategies for controlled regulation of CAR-T cell activity. Schematic representation of switch-off CAR designs, including early suicide gene-based systems and more recent reversible control strategies. **(A)** Inducible caspase-9 suicide switch activated by a small-molecule dimerizer. **(B)** HSV-TK suicide gene system triggered by prodrug administration. **(C)** Antibody-mediated elimination of CAR-T cells via co-expressed surface safety markers (CD20 or truncated EGFR). **(D)** Pharmacologic inhibition of proximal CAR signaling to achieve transient and reversible CAR-T cell inactivation.

One of the earliest and most extensively studied switch-off strategies in CAR-T cell therapy relies on suicide gene systems, with inducible caspase-9 (iCasp9) being the most clinically advanced example. In this approach, CAR-T cells are engineered to express a modified human caspase-9 that can be rapidly activated by a small-molecule dimerizer, thereby inducing synchronized apoptosis in the engineered CAR-T cells. Compared with earlier viral suicide genes, iCasp9 offers faster activation kinetics, reduced immunogenicity, and more predictable *in vivo* performance, as explained in **Figure 3**. Clinically, the safety and efficacy of this system were validated in Phase I trials (e.g., NCT01822652), in which rimiducid administration eliminated more than 90% of circulating CAR-T cells within 30 minutes, thereby halting severe neurotoxicity while allowing a subset of cells to persist for long-term surveillance (42).

Another suicide switch is the Herpes simplex virus thymidine kinase (HSV-TK) system, in which HSV-TK converts the prodrug ganciclovir (GCV) into a toxic nucleotide analog that inhibits DNA synthesis and thereby selectively eliminates dividing CAR-T cells (43). This strategy provides a crucial safeguard against life-threatening complications, including CRS, neurotoxicity, and graft-versus-host disease, and has demonstrated a favorable safety profile in multiple adoptive cell therapy studies (44). In pre-clinical solid tumor models, such as small-cell lung cancer xenografts, GM2 ganglioside-targeted CAR-T cells engineered to express IL-7 and CCL-19 (anti-GM2-7×19) were infused with an HSV-TK safety switch, demonstrating excellent therapeutic efficacy following GCV administration. GCV treatment markedly reduced the circulating CAR-T cells within peripheral blood and reduced the tumor growth, confirming the functional on-demand ablation of the therapeutic CAR-T cells (45). However, a primary translational limitation of the HSV-TK system is its immunogenicity, as the viral origin of the enzyme can trigger host immune responses that prematurely clear CAR-T cells. Furthermore, because it targets DNA synthesis, its “kill speed” is slower than the direct apoptotic mechanism of iCasp9, which may be a critical failure mode in hyper-acute CRS cases (26, 46).

Antibody-mediated switch-off strategies offer an alternative means of external control. In cetuximab-based systems, CAR-T cells are engineered to express receptors that recognize the Fc region of cetuximab, which binds to the epidermal growth factor receptor (EGFR) on tumor cells (47, 48). In this design, CAR-T cell activation is triggered only when cetuximab bridges CAR-T cells to tumor cells, allowing clinicians to finely tune the intensity and duration of CAR-T cell activity by adjusting cetuximab dosing (49). Clinical relevance of this approach was demonstrated in a study of anti-CD5 CAR-T cells in which a truncated EGFR (tEGFR) safety switch was incorporated. Cetuximab administration eliminated more than 90% of the circulating CAR-T cells, and the remaining CAR-T cell population retained sufficient functional capacity to maintain the antitumor surveillance, indicating that antibody-mediated depletion can reduce toxicity without completely abolishing the therapeutic efficacy; therefore, this approach enables the controlled reduction rather than the complete termination of CAR-T cell activity (50).

A related antibody-mediated safety strategy exploits CD20 as an inducible elimination marker on CAR-T cells, enabling their depletion using the FDA-approved anti-CD20 antibody rituximab. Similar to the tEGFR-cetuximab system, this approach enables external pharmacologic control of CAR-T cell persistence, rather than relying on irreversible intracellular suicide mechanisms. In preclinical and early translational studies, CD20-expressing CAR-T cells retained full antitumor activity during the therapeutic phase and were subsequently cleared following rituximab administration to mitigate excessive toxicity (51). However, in contrast to the more rapid debulking observed with cetuximab-mediated clearance, rituximab-induced CAR-T cell elimination depends on immune effector mechanisms, such as antibody-dependent cellular cytotoxicity and complement activation, which may delay the onset of CAR-T cell depletion (52). While these systems benefit from FDA-approved antibodies, their dependence on the host’s immune system introduces significant variability; in patients who are heavily pretreated or lymphodepleted, the ADCC response may be insufficient to achieve rapid clearance.

Critically, while switch-off systems offer a vital safety net, they face a shared economic and therapeutic hurdle: the “reset cost.” Activating a suicide switch often results in the permanent loss of an expensive, personalized cell product, requiring the patient to undergo a new round of manufacturing if the cancer recurs. Balancing this desire for immediate safety with the need for durable antitumor activity remains a primary challenge for clinical implementation (21).

### 2.3 Spatiotemporally controlled ‘Switch on’ CARs

To improve safety while preserving antitumor potency, switch-on CAR systems have been developed to restrict CAR-T cell activation to defined spatial, temporal, or environmental cues. Among these, optogenetic approaches enable external control of CAR signaling via light-sensitive molecular modules. Specifically, systems utilizing the Avena sativa-derived LOV2 domain are triggered by blue light (450–490 nm), which induces a conformational change in the Jα helix to allow for reversible domain docking (53). Such spatiotemporal precision is particularly attractive for limiting off-tumor toxicity in solid tissues while maintaining robust local immune activation (53, 54).

The light-inducible CAR (LiCAR) system exemplifies this strategy by physically separating key CAR signaling components, which reassemble only upon blue-light exposure (55). The system relies on a light-sensitive interaction between two components: a bacterial small stable RNA A (SsrA) peptide fused to a light-oxygen-voltage 2 (LOV2) domain and its binding partner, stringent starvation protein B (SspB). Under dark conditions, the LOV2 domain maintains a conformation that keeps SsrA and SspB apart. However, when illuminated with blue light, LOV2 undergoes a structural change that allows SsrA to bind to SspB. This binding event reassembles the split CAR domains, thereby restoring CAR functionality and triggering T cell activation (56–58). Despite the extreme precision of optogenetic circuits, they remain in the early preclinical stage due to significant translational failure modes. A primary hardware constraint is the limited tissue penetration of blue light (450–490 nm), which necessitates invasive fiber-optic implants or complex upconversion nanoparticles to reach deep-seated solid tumors. Additionally, these purely light-controlled systems may exhibit “leakiness”, low-level background activity due to inadvertent exposure to ambient light or thermal fluctuations, highlighting the need for additional regulatory safeguards (59).

**Figure 4:**
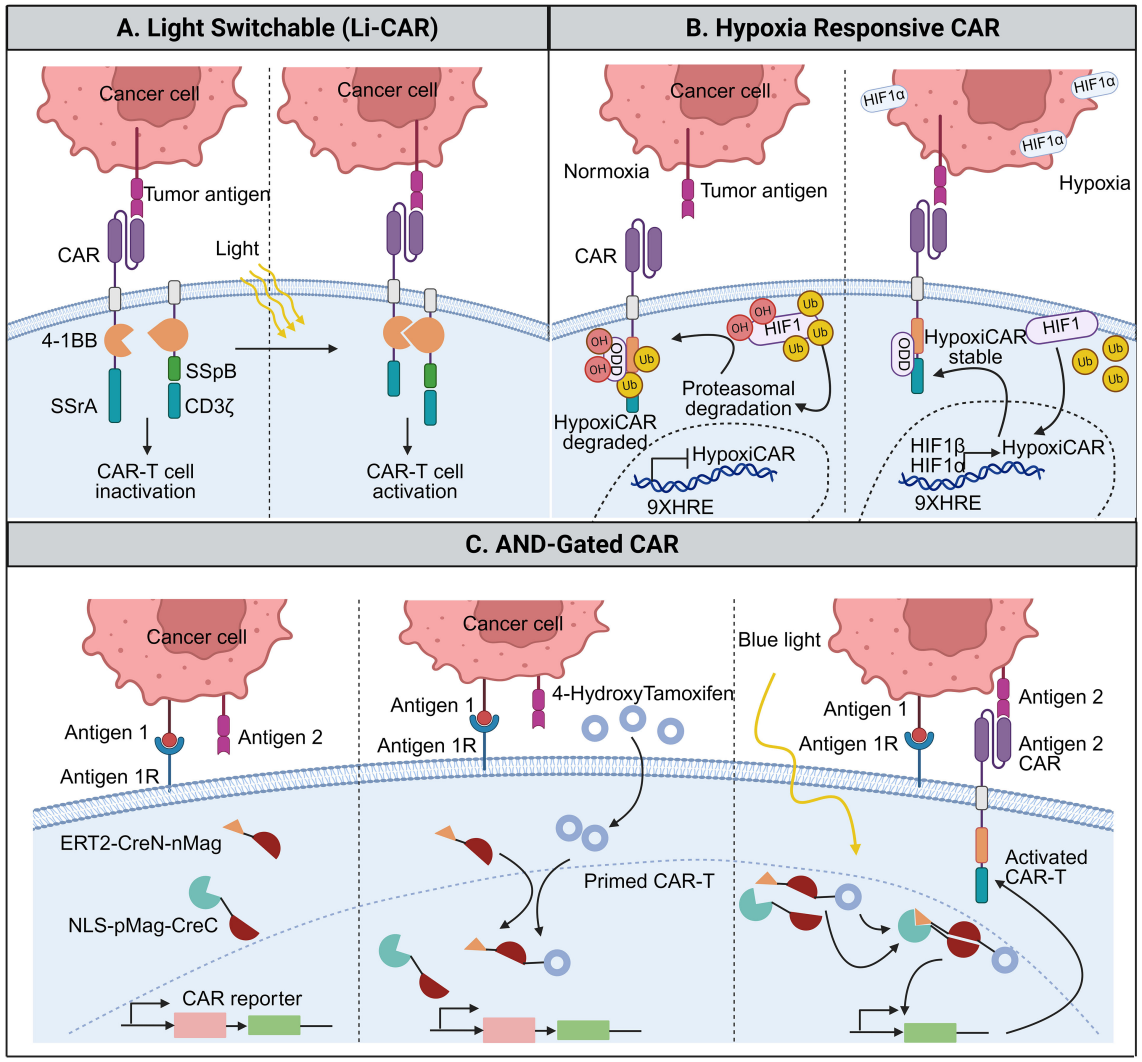
Switch-on CAR systems enabling conditional and localized CAR-T cell activation. Schematic overview of representative strategies in which CAR-T cell activity is selectively enabled by defined environmental or external cues. **(A)** LiCAR remain inactive under dark conditions and are transiently activated upon blue-light exposure through reversible optogenetic domain interactions, allowing spatial control of signaling. **(B)** Hypoxia-responsive CARs restrict CAR expression and/or stability to low-oxygen tumor regions via hypoxia-dependent transcriptional and post-translational regulation, thereby limiting activity in normoxic tissues. **(C)** AND-gated CAR systems require the simultaneous presence of multiple inputs-such as dual antigens combined with exogenous signals (e.g., tamoxifen and light)-to trigger CAR expression or activation, enhancing specificity and reducing unintended targeting. Together, these designs illustrate complementary approaches for achieving precise temporal, spatial, and contextual control of CAR-T cell function.

To address this limitation, multi-input control systems, such as the TamPA-Cre platform, introduce an additional layer of regulation by integrating optical and pharmacological signals. This dual-control design operates as an AND-gated system, requiring simultaneous blue-light exposure (optical signal) and tamoxifen (pharmacological signal) administration to initiate CAR expression at the desired time and place (59). The system uses a tamoxifen-dependent mutant estrogen receptor ligand-binding domain T2 (ERT2), which is fused to one of the two halves of a split Cre recombinase (ERT2-CreN-nMag). In the absence of tamoxifen, the ERT2 domain retains its Cre half in the cytoplasm, keeping the system inactive. A second split Cre half (NLS-pMag-CreC), which contains a nuclear localization sequence (NLS) and is already in the nucleus. The two Cre halves, CreN and CreC, are fused to interacting photoactivatable domains called nMag and pMag, which are derived from a fungal photoreceptor. When the system is primed with tamoxifen, blue light stimulation causes the nMag and pMag domains to dimerize (bind to each other), bringing the two Cre halves together to reconstitute a functional Cre recombinase (59, 60). The very first study using this system in the context of CAR-T cell therapy was published in 2019 by Wu et al, who demonstrated that this strategy enabled highly localized CAR-T cell activation, thereby reducing the risk of on-target, off-tumor toxicity in surrounding healthy tissues (59). Such combinatorial control underscores the value of layered regulatory logic in enhancing the precision of CAR-T cells **Figure 4**.

Beyond externally applied signals, endogenous tumor-associated cues have also been exploited to achieve switch-on control, such as a hypoxia-responsive CAR-T cell system. In this system, CAR expression is controlled by a tightly regulated hypoxia-sensing safety switch that prevents on-target, off-tumor CAR-T cell activation while enabling potent tumor-specific cytotoxicity (50). Hypoxia-sensitive CAR-T cells are engineered to function only under low-oxygen conditions (characteristic of solid tumors), thereby limiting CAR expression or signaling in normoxic healthy tissues and reducing off-target toxicity (61). Mechanistically, hypoxia-responsive CAR-T cells often incorporate hypoxia-responsive elements (HREs) that are activated upon stabilization of hypoxia-inducible factor 1α (HIF1α) in low-oxygen environments. The binding of HIF1α to HREs initiates CAR transcription, thereby restricting CAR-T cell activation to hypoxic tumor regions and sparing well-oxygenated normal tissues (62, 63).

The HypoxiCAR platform further refines this concept through dual oxygen-dependent regulation, combining hypoxia-driven CAR transcription with oxygen-dependent degradation (ODD) of the CAR protein under normoxic conditions. While conceptually elegant, the feasibility of hypoxia-sensing CARs depends on the absolute specificity of the hypoxic gradient; potential “leakiness” in non-tumor hypoxic zones (e.g., in inflamed or ischemic healthy tissue) represents a critical safety concern for clinical implementation (62).

## 3 TME remodeling and microenvironment engineering

Despite the transformative success of CAR-T cell therapy in hematological malignancies, its efficacy in solid tumors remains profoundly constrained by the immunosuppressive and structurally complex tumor microenvironment. The TME constitutes a multifaceted barrier that actively impairs CAR-T cell infiltration, persistence, and effector function, ultimately driving functional exhaustion. Key inhibitory cells include regulatory T cells (Tregs) and myeloid-derived suppressor cells (MDSCs), which suppress antitumor immunity through both contact-dependent mechanisms and the secretion of inhibitory cytokines. As well as elevated levels of soluble immunosuppressive mediators, such as transforming growth factor-β (TGF-β) and interleukin-10 (IL-10), together with high expression of immune checkpoint ligands, including programmed cell death ligand 1 (PD-L1), further attenuate CAR-T cell activation, proliferation, and cytotoxicity (26, 64).

Not only immunosuppression but also adverse metabolic and biophysical features of the TME, such as hypoxia, nutrient deprivation, and acidic pH, impose additional constraints on CAR-T cell survival and function. These hostile conditions disrupt T cell metabolism and signaling, contributing to reduced durability and diminished antitumor activity in solid tumors compared with hematological settings (65–67). These barriers underscore the need for combinatorial strategies that can reshape the TME rather than relying solely on intrinsic CAR design. To overcome these multifaceted constraints, current engineering efforts are shifting from simple descriptions of TME barriers to the development of strategies grounded in three core mechanistic principles: (1) biochemical preconditioning of the TME, (2) autocrine/paracrine signaling support, and (3) biophysical matrix disruption. In this context, combining CAR-T cell therapy with auxiliary agents, such as engineered nanomaterials and oncolytic viruses (OVs), has emerged as a promising tool to modulate the TME, enhance CAR-T cell trafficking and persistence, and restore antitumor immune activity (68, 69). While these hybrid platforms offer potent therapeutic synergy, their transition to the clinic is moderated by significant manufacturing hurdles, including the need for large-scale GMP production and the mitigation of potential autoimmune-like toxicities arising from hyper-activated immune responses.

### 3.1 Nanomaterials for TME modulation

Nanomaterials have emerged as versatile tools to enhance CAR-T cell therapy in solid tumors by modulating the TME, improving T cell persistence, and enabling localized delivery of immunomodulatory agents. Rather than functioning through a single mechanism, nanotechnology-based strategies can be broadly categorized into three mechanistic classes: (i) TME preconditioning, (ii) immune activation via stress induction, and (iii) cell-associated delivery systems, each addressing distinct barriers to CAR-T efficacy (70).

TME preconditioning through targeted delivery of immunomodulators represents one of the most direct applications of nanotechnology. Tumor-targeting liposomes functionalized with the iRGD peptide have been engineered to co-deliver phosphoinositide 3-kinase (PI3K) inhibitors and the immune agonist α-galactosylceramide (α-GalCer) (71), resulting in selective depletion of immunosuppressive cell populations alongside enhanced antitumor immune activation. Similarly, liposomal delivery of TGF-β receptor inhibitors targets a central immunosuppressive axis within the TME. When conjugated to T cell surface markers such as CD90 or CD45, these systems enable spatially restricted drug release at the tumor site, thereby preserving CAR-T cell functionality while minimizing systemic toxicity (72). Collectively, these approaches highlight a shared principle: nanomaterials enhance CAR-T efficacy by locally neutralizing dominant inhibitory pathways within the TME.

A second mechanistic class involves the induction of immunogenic cell death (ICD) and inflammatory reprogramming. Nanozymes, engineered nanomaterials with enzyme-like catalytic activity, can reshape the tumor immune landscape through oxidative stress and photothermal effects (73, 74). For instance, Copper (Cu)-based nanoparticles, which act as nanozymes, enhance treatment by employing photothermal therapy (PTT) to increase reactive oxygen species generation (75, 76). This combined action triggers immunogenic cell death, a process that releases TAAs, subsequently activating dendritic cells (DCs) and stimulating a robust adaptive effector T cell response (76). Hyaluronic acid (HA)-modified Copper sulfide nanoparticles (termed PHCNs) were used to overcome the immunosuppressive TME in B7-H3 CAR-T cell therapy for non-small cell lung cancer (NSCLC). In preclinical models, nanozyme-mediated TME modification and immune priming doubled the rate of complete tumor eradication and significantly prolonged overall survival in mice compared with B7-H3 CAR-T monotherapy. This nanozyme-mediated reprogramming effectively converted the “cold” immunosuppressive TME into an “inflamed” immune-permissive environment, thereby overcoming a critical limitation of solid tumor CAR-T cell therapy (77). Unlike passive drug delivery systems, these nanozymes act as active immunomodulators, initiating endogenous antitumor immunity upstream of CAR-T engagement.

A third strategy focuses on direct integration of nanomaterials with CAR-T cells to create functional biohybrids. Nanomaterials are directly attached to CAR-T cells to enable simultaneous tumor targeting, imaging, and modulation of the TME. Using biorthogonal conjugation, indocyanine green nanoparticles (INPs) were attached to CAR-T cells to generate CAR-T-nanoparticle biohybrids (CT-INPs) (75, 78). Upon localized laser irradiation, these constructs produced mild photothermal effects that disrupted the extracellular matrix (ECM), enhanced vascular permeability, and promoted chemokine secretion, collectively improving CAR-T cell infiltration without impairing viability or cytotoxicity (75, 78, 79). This approach exemplifies a shift from systemic modulation to cell-guided, spatially precise therapeutic delivery.

In parallel, Nanomaterials have also been used to address challenges in cytokine delivery, a critical limitation for adoptive cell therapy. While cytokines such as IL-15 and IL-21 are essential for T cell expansion and persistence, systemic administration is associated with significant toxicity (80, 81). Lipid nanoparticles (LNPs) (approx 300nm), decorated with malemide groups covalently attached to the thiol group on T cell membrane, provide a sustained, pseudo-autocrine cytokine supply, enhancing CAR-T cell durability and antitumor efficacy without detectable systemic adverse effects (82). Similarly, CAR-T cells have been used as carriers for IL-12-loaded nanochaperones that rapidly degrade upon T cell activation (when high levels of thiol groups are encountered), releasing IL-12 to recruit additional immune cells and amplify the antitumor response (83). These systems highlight a key advantage of nanotechnology: the ability to decouple cytokine efficacy from systemic exposure through localized and stimulus-responsive delivery.

**Figure 5:**
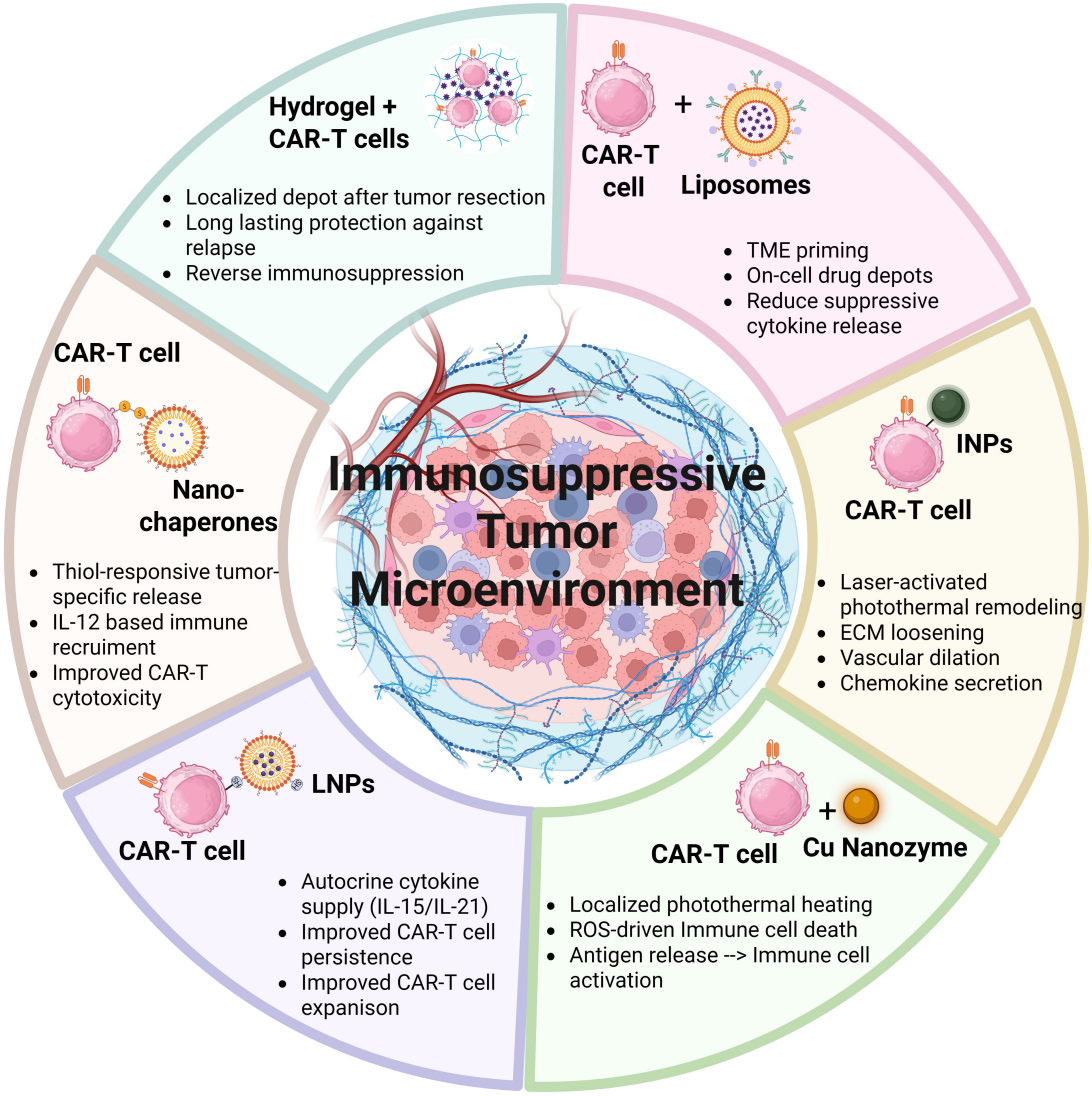
Nanomaterial-enabled strategies to remodel the tumor microenvironment and enhance CAR-T cell therapy. Schematic representation of complementary nanomaterial-based approaches designed to overcome immunosuppressive features of TME and improve CAR-T cell efficacy in solid tumors. Nanomaterials are used either in combination with or directly coupled to CAR-T cells to achieve localized immunomodulation. Shown strategies include: liposome-mediated TME priming and localized drug release to attenuate suppressive cytokine signaling; photothermal nanoparticle-CAR-T biohybrids that promote extracellular matrix remodeling, vascular dilation, and chemokine secretion; copper-based nanozymes that induce immunogenic tumor cell death and secondary immune activation**;** lipid nanoparticle systems providing pseudo-autocrine cytokine support to enhance CAR-T cell expansion and persistence; nanochaperone “backpacks” enabling activation-responsive cytokine release, and hydrogel-based depots for confined CAR-T cell delivery and sustained local immune support.

Despite their promises, cell-associated nanoparticle systems face limitations. Since the nanoparticle payload does not expand as T cells proliferate *in vivo*, the amount of drug carried per cell becomes diluted across successive cell divisions. Furthermore, these backpacks generally lack dynamic regulation, limiting their ability to adjust drug release in response to changes in TME. To address these challenges, localized delivery platforms such as injectable hydrogels have been developed. For example, a hyaluronic acid (HA) hydrogel encapsulating CAR-T cells, IL-15 nanoparticles, and anti-PD-L1-loaded platelets enabled sustained local immunomodulation following tumor resection, significantly enhancing CAR-T cell activity and reducing tumor recurrence in preclinical models, as shown in **Figure 5** (84). Collectively, these findings demonstrate that nanomaterials enhance CAR-T therapy through complementary yet distinct mechanisms ranging from blockade of inhibitory pathways and immune priming to physical remodeling of the tumor stroma and controlled cytokine delivery. Understanding these mechanistic differences is essential for the rational design of next-generation combinatorial strategies.

### 3.2 Oncolytic viruses as inflammatory scaffolds

Oncolytic viruses are either naturally occurring or genetically engineered viruses that selectively infect and replicate within malignant cells while leaving normal tissue unharmed (85). Beyond their direct oncolytic activity, OVs serve as multifunctional immunomodulatory platforms that enhance CAR-T cell efficacy through interconnected mechanisms. These can be broadly categorized into (i) immune priming via tumor lysis, (ii) provision of auxiliary T cell activation signals, (iii) remodeling of physical and immunosuppressive barriers, and (iv) localized delivery of immunostimulatory payloads.

A central mechanism of OV activity is immune priming through immunogenic tumor cell lysis. Viral replication within tumor cells leads to cell rupture and the release of tumor-associated antigens (TAAs) and danger-associated molecular patterns (DAMPs), thereby promoting dendritic cell activation and antigen presentation. This process effectively converts immunologically “cold” tumors into inflamed, immune-permissive environments, thereby enhancing CAR-T cell recruitment and function (86–88). Unlike nanomaterial-based systems, which primarily deliver exogenous modulators, OVs initiate endogenous immune activation through biological amplification.

In addition to priming, OVs can provide auxiliary activation signals to CAR-T cells, particularly through engagement of the native T cell receptor (TCR). Viral or virally encoded antigens can stimulate TCR signaling in CAR-T cells, complementing CAR-mediated activation. In immunocompetent models of melanoma and glioma, systemic OV administration enhanced CAR-T cell proliferation, effector function, and memory differentiation via this dual signaling mechanism (89). Preloading CAR-T cells with viruses such as vesicular stomatitis virus (VSV) or reovirus further enabled *in vivo* expansion and reactivation through viral boosting. This capacity to deliver both antigenic and inflammatory stimulation highlights a key distinction from nanomaterials, which generally lack intrinsic antigenicity (89).

OVs also play a critical role in remodeling physical and stromal barriers within the TME. A recombinant oncolytic vaccinia virus expressing hyaluronidase (Hyal1) was shown to degrade hyaluronic acid within tumors, thereby improving immune cell infiltration and enhancing overall antitumor efficacy (90). This matrix-disruptive capability reduces tumor burden, as dense ECM components restrict CAR-T cell trafficking and persistence.

Another major function of OVs is to reverse immunosuppressive signaling within the TME. Cancer cells shape the TME by actively recruiting Tregs, tumor-associated macrophages (TAMs), MDSCs, and stromal elements that collectively suppress effector T cell function. OVs counteract this suppression by inducing localized inflammation and reshaping cytokine and chemokine gradients, facilitating CAR-T cell infiltration and sustained activity (91, 92). In pancreatic cancer models, an oncolytic adenovirus engineered to express TNF-α and IL-2 (Onc.Ad-TNF-α/IL-2) significantly enhanced the antitumor efficacy of mesothelin-targeted CAR-T cells incorporating a 4-1BB co-stimulatory domain. This improved response was attributed to localized cytokine release of TNF-α and IL-2, combined with direct viral oncolysis, highlighting the complementary roles of viral and CAR-T-mediated cytotoxicity (93).

Importantly, OVs can be engineered as localized delivery vehicles for immunotherapeutic payloads, including cytokines and immune checkpoint inhibitors. This enables high intratumoral concentrations while minimizing systemic toxicity. Shaw et al. developed an oncolytic adenovirus (CAd12-PDL1) that expresses both a PD-L1-blocking mini-antibody and the IL-12p70 cytokine. When combined with HER2-targeted CAR-T cells, this approach effectively suppressed primary tumor growth and metastatic spread, while limiting systemic toxicity associated with systemic checkpoint blockade (26, 94).

**Figure 6:**
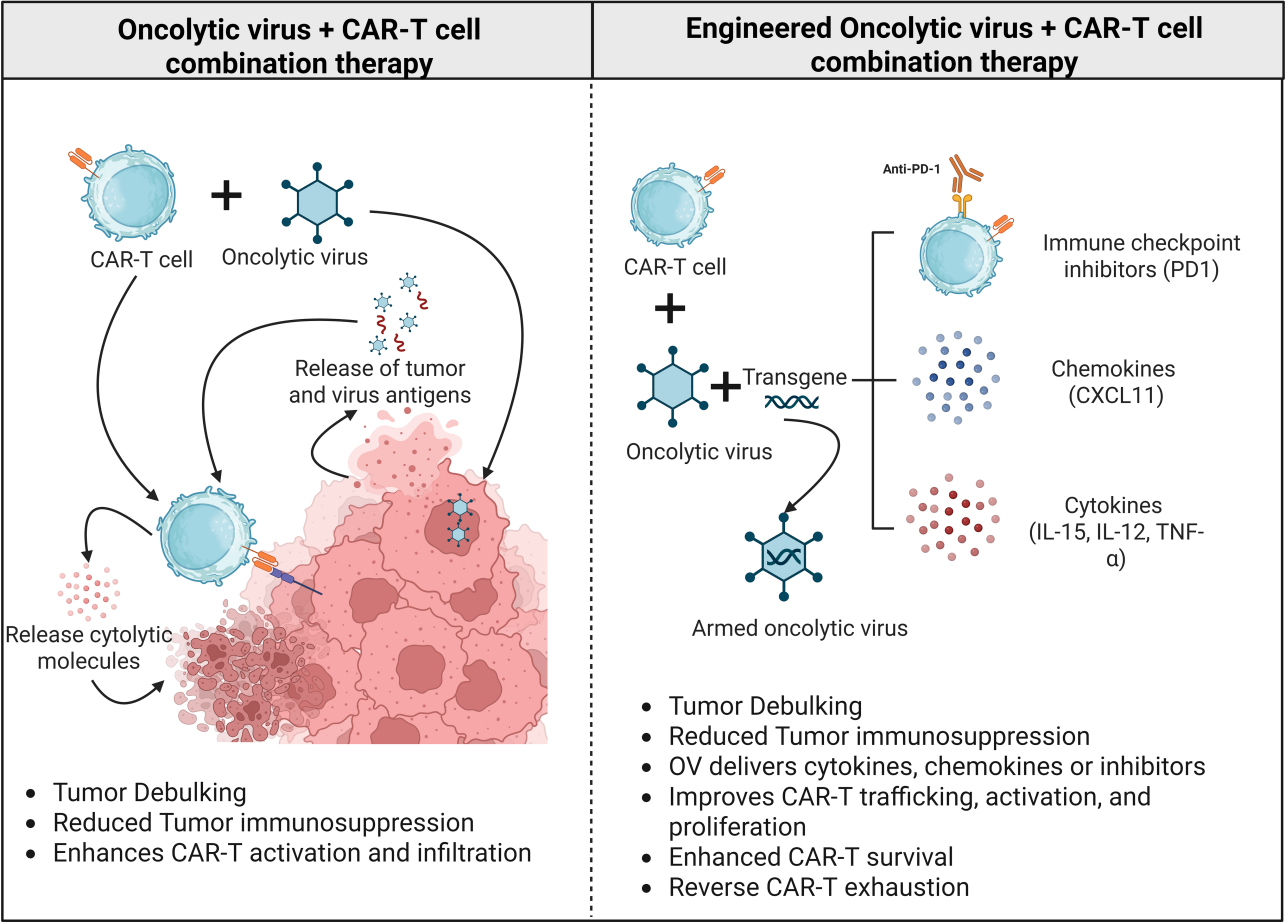
Synergistic mechanisms of oncolytic virus and CAR-T cell combination strategies in solid tumors. Conceptual illustration comparing conventional and engineered oncolytic virus + CAR-T cell combination approaches. (Left) Co-administration of CAR-T cells with unmodified OVs promotes direct tumor debulking through complementary cytolytic mechanisms and induces immunogenic tumor cell death, thereby releasing tumor- and virus-derived antigens that enhance CAR-T cell activation and infiltration. This inflammatory milieu partially alleviates local immunosuppression and supports antitumor immune responses. (Right) Engineered OVs further amplify therapeutic efficacy by delivering immunomodulatory transgenes, including immune checkpoint inhibitors, cytokines, and chemokines, directly within the tumor microenvironment. Localized expression of these factors enhances CAR-T cell trafficking, activation, proliferation, and persistence while reducing exhaustion and suppressive signaling.

From a signaling perspective, effective antitumor T cell responses require the integration of antigen recognition, co-stimulatory signaling, and inflammatory signals. While second- and third-generation CARs are designed to provide signals 1 and 2, the inflammatory “signal 3” is frequently reported to be insufficient in the solid TME. While OVs are potent inducers of type I interferons, the magnitude and duration of this signal 3 support likely vary depending on viral tropism and the baseline inflammatory state of the tumor. This inflammatory signal is typically supplied by cytokines such as IL-12 or type I interferons (IFNs). Type I IFNs are crucial in antiviral and antitumor responses, enhancing the host’s adaptive immune system. Notably, OVs are potent inducers of type I interferons, which play a central role in antiviral immunity and support T cell survival, expansion, and cytotoxicity (95). Type I interferon signaling in CAR-T cells may be amplified through 4-1BB-mediated activation of TNF receptor-associated factor 2 (TRAF2), suggesting that CAR-T products incorporating 4-1BB domains or chimeric co-stimulatory receptors (CCRs) may be particularly well suited for OV-based combination strategies (88). Adjusting the signal-3 pathways, either through OV engineering or CAR design, substantially improves the performance of CAR-T cells once they are infused *in vivo*, as shown in **Figure 6**.

Combining CAR-T cells with nanomaterials or oncolytic viruses (OVs) introduces significant regulatory and manufacturing complexity. The FDA classifies these as “combination products,” requiring separate safety validation for both the cellular and viral/material components, which doubles the cost of clinical trials. The major drawback of Nanomaterials is that the scaling of the production of “cell-surface backpacks” is difficult; ensuring a uniform dose of nanoparticles across billions of T-cells remains a major quality-control challenge. The clinical efficacy of OVs is often limited by the patient’s pre-existing antiviral immunity. Neutralizing antibodies can clear the virus before it can remodel the TME or recruit CAR-T cells, particularly in systemically administered doses.

### 3.3 Mechanistic comparison of nanomaterials and oncolytic viruses in CAR-T therapy

Although nanomaterials and oncolytic viruses represent distinct therapeutic platforms, their functional convergence lies in their ability to reprogram the TME and enhance antitumor immunity through complementary immunological pathways. At a mechanistic level, both strategies promote antigen availability and immune activation, but they differ fundamentally in how these processes are initiated and regulated (96, 97).

Oncolytic viruses operate as dynamic, self-amplifying immunological amplifiers. By inducing immunogenic cell death (ICD), OVs trigger a cascade of endogenous immune activation via type I interferon and STING signaling. This “biological gas” effectively converts “cold” tumors into inflamed, immune-responsive states, but is inherently less controllable and may provoke systemic inflammation or cytokine-driven toxicities (98). In contrast, nanomaterials function as externally programmable, precision delivery vectors. Rather than initiating broad innate activation, they allow for the spatially and temporally controlled tuning of specific axes, such as metabolic fitness or trafficking. While offering more predictable pharmacokinetics, nanomaterials generally lack the self-propagating amplification seen with viral approaches (99).

These mechanistic distinctions translate into divergent system-level behaviors: oncolytic viruses operate as self-propagating immunological amplifiers, whereas nanomaterials act as externally programmable delivery vectors. Consequently, oncolytic viruses may be more effective in converting “cold” tumors into inflamed, immune-responsive states, while nanomaterials are better suited for fine-tuning CAR-T cell function and minimizing off-target effects. However, the strong innate immune activation triggered by viral platforms increases the risk of cytokine-driven toxicities and immunogenic clearance, whereas nanomaterials face challenges related to delivery efficiency, retention, and potential accumulation (98, 100).

Understanding these deeper distinctions provides a framework for rational design: OVs are best suited for initial TME “inflammation,” while nanomaterials offer a means to fine-tune CAR-T function and minimize off-target effects. However, the actual clinical application of either platform remains contingent upon overcoming the economic burden and regulatory complexity associated with “combination products” under GMP standards.

## 4 Genetic engineering for enhanced persistence and potency

Although advances have been made in CAR-T cell design and manufacturing, the therapeutic efficacy and long-term persistence of CAR-T cells remain significant limitations, particularly in treating solid tumors. Various factors, including physical barriers, suppressive cellular components, suppressive cytokines, and inhibitory molecular signals, collectively impede CAR-T cell infiltration, survival, and sustained antitumor activity. Addressing these intrinsic limitations has therefore become a central focus in next-generation CAR-T cell engineering. Advances in genetic manipulation have enabled precise and durable reprogramming of CAR-T cells to resist these suppressive pressures. Genome-editing technologies, particularly the CRISPR-Cas system, allow targeted disruption of inhibitory receptors, transcriptional regulators, and metabolic checkpoints that constrain CAR-T cell function. Additionally, interference-based approaches, such as short-hairpin RNA (shRNA), offer a flexible, tunable strategy to silence genes that promote exhaustion or apoptosis, or that suppress suppressive signaling, in CAR-T cells. These genetic strategies not only enhance immediate cytotoxicity but also rewire CAR-T cell fate decisions, promoting sustained function, improved persistence, and long-term tumor control. In the following sections, we discuss how CRISPR-based genome editing and shRNA-mediated gene silencing are being used to address CAR-T cell exhaustion and durability in the immunosuppressive TME.

### 4.1 CRISPR-Cas9-mediated genome editing

CRISPR-Cas9 genome editing enables permanent genetic modifications by utilizing a guide RNA (gRNA) to direct the Cas9 nuclease to a specific genomic locus, where it induces targeted Double-Strand Breaks (DSBs). These breaks are typically repaired via the error-prone Non-Homologous End Joining (NHEJ) pathway, resulting in gene knockout. However, a critical safety concern arises during multiplexed editing: the simultaneous occurrence of multiple DSBs significantly increases the risk of inter-chromosomal translocations and large-scale genomic deletions, which necessitate rigorous genomic stability assessments in clinical-grade manufacturing (101, 102).

One of the most prominent drivers of CAR-T cell dysfunction in solid tumors is chronic antigen stimulation, which induces sustained expression of inhibitory immune checkpoint receptors, leading to T cell exhaustion. The CRISPR-Cas9 technique has been extensively used by researchers to knock out genes encoding these inhibitory receptors, thereby preventing suppressive signaling and restoring CAR-T cell effector function (103). Using CRISPR to knock out the programmed cell death protein 1 (PDCD1) gene prevents CAR-T cells from receiving inhibitory signals upon encountering PD-L1 on tumor cells. Preclinical studies consistently demonstrate that PD-1-deficient CAR-T cells exhibit enhanced cytokine production (including IFN-γ and TNF-α), increased proliferation, and better tumor control across multiple murine cancer models (104). Early-phase I clinical trials, such as NCT03179943, have also confirmed the feasibility and safety of PD-1-edited CAR-T cells in humans, thus supporting the translational potential of this strategy (105, 106).

Studies have confirmed that PD-1, as well as CRISPR-mediated deletion of other immune checkpoints, has further underscored the potential of this strategy. Deletion of cytotoxic T-lymphocyte-associated antigen 4 (CTLA-4) improves the proliferative capacity, persistence, and antitumor activity of CAR-T cells in preclinical models (107). Inhibitory receptors such as T cell immunoglobulin mucin domain-containing protein 3 (TIM-3), Lymphocyte activation gene 3 protein (LAG-3), and 2B4 (CD244) play distinct, non-redundant roles in driving T-cell exhaustion under chronic stimulation. Using CRISPR-Cas9 to individually disrupt these genes in NY-ESO-1-specific T cells significantly improved resistance to functional exhaustion both *in vitro* and *in vivo* (108).

A significant advantage of CRISPR-Cas9 technology is its ability to simultaneously disrupt multiple genes within a single shot. Ren et al. demonstrated that a single CRISPR-Cas9 system could perform multiplex genome editing to generate universal, allogeneic CAR-T cells by knocking out endogenous TCR and HLA class I genes. The addition of PD-1 disruption further enhanced the antitumor activity of these engineered T cells in mouse models, indicating the synergistic benefit of multiplex genome editing for both immune evasion and functional enhancement (109).

In addition to surface inhibitory receptors, a specific set of transcription factors is also involved in T cell exhaustion (110). Transcription factors such as TOX and the members of the NR4A family (NR4A1, NR4A2, NR4A3) have been identified as central regulators of T cell exhaustion in several chronic stimulation models. Similarly, the manipulation of PRDM1 and TCF1 may promote a precursor exhausted T cell (TPEX) phenotype; however, the stability of this ‘stemness’ once cells are exposed to the immunosuppressive TME remains a subject of ongoing investigation (105). Using multiplex CRISPR editing to simultaneously delete all three NR4A genes, making human CAR-T cells highly resistant to exhaustion after repeated antigen exposure, both *in vitro* and *in vivo*. Comparative analysis revealed that triple-knockout (TKO) cells outperformed single- or double-knockout cells, exhibiting superior persistence, enhanced antitumor activity across multiple donors, and increased mitochondrial oxidative phosphorylation, thereby supporting sustained function within tumors (111). Additionally, targeting genes such as PR domain zinc finger protein 1 (PRDM1) and T cell factor 1 (TCF1) with CRISPR can enhance T cell ‘stemness,’ promoting a precursor-exhausted T cell (TPEX) phenotype that remains responsive to immunotherapy (112).

CRISPR-based editing has also been applied to counteract extrinsic immunosuppressive signals within the TME. TGF-β is a dominant suppressive cytokine in solid tumors. CAR-T cells lacking TGF-β receptor II (TGFBR2) demonstrated significantly enhanced proliferation and tumor-killing capacity *in vitro*, even in the presence of high TGF-β concentrations. *In vivo*, TGFBR2-knockout CAR-T cells showed improved persistence and increased proportions of central and effector memory subsets, both of which are critical for durable antitumor immunity (113). Combining TGFBR2 knockout with PD-1 disruption further enhanced efficacy in highly suppressive TMEs, underscoring the potential of multiplex gene-editing strategies.

Recent advances in CRISPR technology are further expanding its therapeutic potential. Double-strand break-free (DBS) approaches, including base editing and prime editing, enable precise genetic modifications with reduced genotoxicity and improved safety profiles (114). So, CRISPR-Cas9-mediated multiplex knockouts of inhibitory receptors, transcriptional exhaustion drivers, and suppressive cytokine pathways offer a powerful strategy to overcome both intrinsic and extrinsic resistance mechanisms in CAR-T cell therapy. Integrating advanced gene-editing strategies into next-generation CAR-T cell therapies can improve treatment efficacy, reduce off-target effects, and increase accessibility through cost-effective, targeted interventions (115).

### 4.2 shRNA technology for tunable silencing

Short-hairpin RNA (shRNA) technology provides a post-transcriptional gene silencing mechanism that does not alter the underlying genomic DNA. Once expressed, shRNA is processed by the Dicer enzyme into small interfering RNAs (siRNAs), which are loaded into the RNA-induced silencing complex (RISC) to mediate the sequence-specific degradation of target mRNA. This mRNA-level interference enables graded, reversible suppression of inhibitory receptors, offering a ‘tunable’ alternative to the permanent, binary nature of CRISPR-mediated knockouts. Short-hairpin RNA technology enables stable yet reversible gene silencing by targeting specific mRNAs for degradation, offering a regulatory flexibility that is particularly advantageous when complete gene knockout may compromise CAR-T cell viability or induce unintended toxicities (116). Unlike CRISPR-based permanent genome editing, shRNA enables graded suppression of inhibitory pathways, making it well-suited for fine-tuning CAR-T cell function within the dynamic and immunosuppressive TME. This approach is especially relevant in solid tumors, where excessive genetic disturbance may impair exhaustion or persistence.

A major advantage of shRNA engineering is the ability to intrinsically silence immune checkpoint receptors within CAR-T cells, thereby enhancing activation, persistence, and cytotoxicity without the systemic toxicities associated with checkpoint-blocking antibodies (117). Our group and others have demonstrated that shRNA-mediated suppression of negative regulatory pathways can substantially improve CAR-T cell performance in both hematological and solid tumor models (116, 118–120). For example, adrenergic stress is a key but underappreciated immunosuppressive axis in the solid TME. Beta-2 adrenergic receptor (ADRB2) functions as a stress-induced checkpoint receptor on T and NK cells, reducing antitumor immunity in catecholamine-rich tumor microenvironment (67, 121). Using shRNA technology, we developed ADRB2-downregulated CAR-T cells that are protected from adrenergic stress signaling. Incorporation of ADRB2 shRNA into second-generation CAR constructs significantly enhanced T cell activation and cytokine secretion. Importantly, these cells exhibited increased glucose transporter 1 (GLUT-1) expression and elevated Peroxisome proliferator-activated receptor gamma coactivator 1α (PGC1-α) levels, reflecting improved metabolic fitness and mitochondrial biogenesis, which translated into superior antitumor activity *in vitro* against prostate and colorectal cancer models (67, 119). In another study, shRNA-mediated downregulation of the epigenetic checkpoint histone deacetylase 11 (HDAC11) enhanced CAR-T cell proliferation, reduced exhaustion markers (PD-1 and TIM-3), and promoted central memory T cell (TCM) differentiation via upregulation of Eomes (118). HDAC11-silenced NKG2D-CAR-T cells achieved improved tumor control and prolonged survival *in vivo* compared with conventional CAR-T cells (118). Similarly, other well-known immune checkpoints, such as PD-1, TIM-3, and LAG-3, have been intrinsically inhibited in CAR-T cells using shRNA technology, thereby enhancing CAR-T cell activity in preclinical studies (105, 122, 123).

However, a central limitation of single-gene silencing is the redundancy of checkpoints and compensatory upregulation. Tumors can adapt to the inhibition of one pathway by engaging alternative inhibitory receptors, thereby restoring immune suppression (124). To address this, current shRNA strategies are shifting toward multiplexed checkpoint silencing, which simultaneously silences 2–4 immune checkpoint genes. A novel dual-function lentiviral vector was developed to enable efficient shRNA-mediated silencing of both PD-1 and T-cell immunoreceptor with immunoglobulin and immunoreceptor tyrosine-based inhibitory motif domains (TIGIT) while maintaining strong CAR expression in CD19-CAR-T cells. These CAR-T cells, designated CRC01, demonstrated significantly enhanced antitumor activity both *in vitro* and *in vivo* compared with conventional CAR-T cells. Upon CD3/CD28 stimulation, analysis of CRC01 cells showed that PD-1 and TIGIT expression were markedly lower in CAR-positive T cells than in their CAR-negative counterparts, confirming effective, targeted knockdown of both immune checkpoint genes (125).

Advancing multiplex shRNA delivery introduces technical and biological challenges. Combining multiple shRNAs within a single vector and scaffolds that facilitate co-expression with therapeutic transgenes presents a promising strategy for creating CAR-T cells with customized phenotypes. However, efforts to multiplex shRNAs using the clinically approved miR196a2 scaffold have been linked to reduced vector titers. The reductions in vector titer varied among the tested duplex and triplex shRNA constructs; however, the overall titers remained too low for clinical application. To overcome this issue, a proprietary scaffold was developed to support the expression of duplexed and triplexed shRNAs while increasing vector titers by at least 2 to 3 times (126). Further development of the multiplexed shRNA vector system is underway to assess its potential to enhance the therapeutic efficacy of CAR-T cells. In another study, four different shRNAs were expressed in a single vector, although the optimization remains ongoing (126).

As the number of targeted genes increases, careful systems-level evaluation becomes essential. Multiple immune checkpoints often converge on shared downstream signaling nodes, such as SHP-1 and SHP-2 phosphatases, meaning that not all combinations yield additive benefit. Future shRNA-based CAR-T design must therefore prioritize synergistic target selection, focusing on pathways that regulate distinct yet complementary inhibitory mechanisms. Furthermore, combining shRNA-engineered CAR-T cells with other immunotherapies creates multifaceted strategies that can counteract immunosuppression, enhance T-cell recruitment and survival, and boost cytotoxic and memory responses. This approach is particularly promising for treating solid tumors, where standalone CAR-T cell therapy has faced challenges owing to physical barriers, antigen heterogeneity, and T-cell exhaustion.

Furthermore, unlike CRISPR, shRNA relies on the cell’s internal RNAi machinery; overloading this machinery with multiple shRNA sequences can saturate the Exportin-5 and Dicer pathways, interfering with natural microRNA processing and leading to cellular stress or reduced CAR-T persistence. Additionally, because shRNA constructs in certain viral vectors do not consistently achieve stable, high-level expression in all daughter cells, “payload dilution” during rapid T-cell expansion can be a failure mode that may result in the loss of the engineered phenotype over time (127). The comparison between these strategies is explained in **Table 1**.

**Table 1:** Risk-benefit comparison of genome engineering platforms in CAR-T cells.

Feature	CRISPR-Cas9 (permanent KO)	shRNA (tunable KD)
Genomic impact	Permanent DNA alteration	Post-transcriptional (mRNA) only
Primary safety risk	Chromosomal translocations; DSBs	Saturation of RNAi machinery (Dicer/Exportin-5)
Stability of effect	Absolute (Inherited by daughter cells)	Potentially transient due to “payload dilution.”
Control logic	Binary (Complete on/off)	Graded (rheostatic/tunable)
Best clinical use case	Allogeneic TCR/HLA deletion	PD-1 or metabolic checkpoint tuning
Vector burden	High (due to Cas9/gRNA size)	Moderate (Multiple cassettes may reduce titer)

### 4.3 CRISPR-based knockout versus shRNA-mediated knockdown

Genome modulation strategies in CAR-T cells broadly fall into two paradigms: permanent gene disruption (e.g., CRISPR/Cas-mediated knockout) and tunable gene suppression (e.g., shRNA-mediated knockdown) (116). While both approaches aim to enhance CAR-T cell efficacy by attenuating inhibitory pathways or reprogramming cellular function, they differ substantially in durability, controllability, safety profile, and manufacturing implications, which directly influence their suitability for clinical translation.

The main advantage of CRISPR/Cas-mediated knockout is the complete removal of undesired signals. But in the context of CAR-T cell therapy, both CAR expression and genome-editing efficiencies are incomplete, meaning not all CAR cells can be assumed to be edited, resulting in a heterogeneous therapeutic product. Additionally, complete knockdown of one gene can lead to overexpression of some other inhibitory gene as a compensatory mechanism. Double-strand DNA breaks can introduce off-target mutations, chromosomal rearrangements, and genotoxic stress, while incomplete editing results in product heterogeneity, complicating both functional predictability and regulatory evaluation (128). These risks are amplified in multiplex editing strategies, which are increasingly explored to overcome redundant immunosuppressive pathways in solid tumors. Moreover, the requirement for high editing efficiency and stringent genomic quality control introduces a significant manufacturing burden, potentially limiting scalability (127).

On the other hand, because shRNA and CAR are expressed from the same vector, we can ensure that all CAR-positive cells also express shRNA. shRNA provides incomplete knockdown, which can be advantageous in some cases, for example, where complete ablation may lead to unintended consequences such as impaired CAR-T persistence, dysregulated activation, or toxicity. The incorporation of multiple shRNA cassettes into CAR constructs increases vector complexity, raising concerns about transcriptional interference, competition for shared processing machinery, and reduced long-term expression stability. Furthermore, many inhibitory pathways in T cells converge on shared downstream signaling nodes; therefore, simultaneous knockdown of multiple receptors may not yield additive functional gains (129).

From a therapeutic design perspective, the choice between knockout and knockdown should be context-dependent. Permanent gene knockout is generally preferred when sustained, complete pathway abrogation is required, particularly for targets that exert dominant, non-redundant inhibitory effects on CAR-T cell function. This approach is most suitable in settings such as solid tumors with chronic antigen exposure, where durable resistance to immunosuppression is essential. It is especially advantageous when the target gene is dispensable for core T cell fitness, thereby minimizing risks to proliferation and persistence. However, its application is best justified in high-risk or refractory disease contexts where enhanced potency outweighs the added manufacturing complexity and regulatory burden associated with genome editing.

In contrast, tuneable gene knockdown is more appropriate for targets with pleiotropic or context-dependent functions, where complete ablation may compromise CAR-T cell stability or safety. Partial suppression allows preservation of basal signaling required for T cell homeostasis while still enhancing effector function. This approach is particularly valuable in tumors with heterogeneous antigen expression, where fine control of activation thresholds can reduce the risk of on-target/off-tumor toxicity. Additionally, knockdown strategies offer greater flexibility and are often favored when manufacturing simplicity and minimization of genomic risk are key considerations.

## 5 Strategies to improve CAR-T trafficking and infiltration

Effective antitumor activity of CAR-T cells requires not only potent cytotoxicity but also efficient trafficking to and infiltration into tumor sites. In solid tumors, this process is frequently impaired by dysregulated chemokine signaling. Many tumors either downregulate T cell-attracting chemokines or preferentially secrete chemokines that recruit immunosuppressive populations rather than effector lymphocytes, resulting in poor CAR-T cell homing (130). Unlike hematologic malignancies, solid tumors are embedded within a highly structured and physically restrictive microenvironment. Dense extracellular matrix deposition, aberrant vasculature, and extensive networks of tumor-associated fibroblasts collectively form substantial barriers that limit T cell extravasation and intratumoral migration (131). Even when CAR-T cells reach the tumor periphery, these structural constraints can prevent deep penetration into the tumor core, thereby limiting immune activity and incomplete tumor eradication. Addressing these trafficking and infiltration deficits has therefore emerged as a critical priority for extending CAR-T cell therapy to solid malignancies. Current strategies aim to reprogram CAR-T cells to better sense and respond to tumor-derived chemokines and to remodel the tumor stroma and vasculature to facilitate immune cell entry. In the following subsection, we focus on chemokine-based CAR-T cell engineering strategies to enhance tumor homing, infiltration, and distribution within solid tumors.

### 5.1 Chemokine-based CAR-T cell advancements

Efficient trafficking of CAR-T cells to solid tumor sites remains a significant barrier to therapeutic success. Preclinical studies consistently show that only a tiny fraction of the transferred T cells, often around 1-2%, actually enter the tumor mass. In a prostate cancer model using PSMA-targeted CAR-T cells, fewer than 0.2% of transferred cells were detected within the tumor, while the majority accumulated in non-target organs such as the thyroid and salivary glands (130, 132). Optimizing CAR-T cell homing not only enhanced antitumor activity but also allowed dose reduction and minimized off-tumor toxicity.

Chemokine gradients orchestrate T cell migration, yet solid tumors frequently disrupt this system by suppressing effector T cell-attracting chemokines or by producing chemokines that preferentially recruit immunosuppressive populations. Rather than facilitating immune surveillance, distorted chemokine signals promote the accumulation of Tregs, MDSCs, and M2-type TAMs, effectively excluding cytotoxic lymphocytes from the tumor core (133). One strategy to overcome this mismatch is to engineer CAR-T cells to express chemokine receptors that bind ligands enriched in the TME, thereby restoring chemotactic responsiveness and enhancing intratumoral accumulation (134).

CXCL8 (also known as IL-8) is a key chemokine abundantly expressed in many solid tumors, where it is typically associated with inflammation, angiogenesis, and immunosuppression. Although CXCL8-rich environments are generally hostile to CAR-T cell therapy, this axis can be repurposed by engineering CAR-T cells to express its cognate receptors, CXCR1 or CXCR2. This modification converts a protumorigenic signal into a directional cue that enhances CAR-T cell trafficking and retention within tumors (135). In preclinical models, CD70-directed CAR-T cells co-expressing CXCR1 or CXCR2 exhibited markedly improved migration toward IL-8 gradients and tumor-derived supernatants, leading to robust tumor regression and prolonged survival compared with unmodified CAR-T cells (136). Another study in a pancreatic ductal adenocarcinoma (PDAC) model confirmed the benefits of CXCR1/2 engineering across CXCL8-rich malignancies, including improved CAR-T cell survival, reduced accumulation of myeloid suppressor cells, and inhibition of metastasis (137).

Similarly, elevated expression levels of CXCL1 and CXCL2 in several solid tumors have further motivated the use of CXCR1/2-modified CAR-T cells to broaden chemokine responsiveness (138). Both CXCR1- and CXCR2-targeting CAR-T cells demonstrated dose-dependent migration abilities *in vitro* and enhanced tumor localization even 48 hours after CAR-T cell injection (136). Engineering lymphocytes from the tumor ascites of ovarian cancer patients to express CXCR2 enhanced their migration toward both autologous and allogeneic ascites *in vitro*, and the ascites exhibited high levels of CXCL1 and CXCL8 expression (139). CAR-T cells expressing CXCR2 and targeting integrin αvβ6 showed better tumor control in a human pancreatic tumor xenograft mouse model than CAR-T cells lacking CXCR2 (140).

The CXCL13-CXCR5 axis represents another promising route for improving CAR-T cell infiltration. CXCL13 is increasingly recognized as a chemokine enriched in specific solid tumors, where it influences lymphocyte organization and migration. Activation of CXCR5 has been linked to improved calcium signaling and enhanced motility in T cells (141, 142). Osteosarcoma is often overlooked, and our recent study demonstrated that CXCL13 was highly expressed in the 143B and U-2OS osteosarcoma cell lines (143). The co-expression of CXCR5 enhanced the migratory and infiltrative abilities of second-generation NKG2D-based CAR-T cells *in vitro* and in mouse xenografts (143). Similar strategies have been applied in non-small cell lung cancer, where EGFR-targeted CAR-T cells expressing CXCR5 achieved superior tumor infiltration and control without increasing off-tumor toxicity, while maintaining cytotoxic activity comparable to conventional CAR-T cells (144).

An alternative approach focuses on reshaping the chemokine milieu rather than solely modifying receptor expression. CCR7, a receptor critical for naïve and central memory T cell trafficking, has been leveraged in CAR-T cell engineering strategies that secrete CCL19 alongside homeostatic cytokines such as IL-7 (145). CAR-T cells co-expressing IL-7 and CCL19 have demonstrated remarkable efficacy across multiple solid tumor models, including mesothelioma, pancreatic cancer, and glioblastoma. These cells not only achieved superior tumor control and prolonged survival but also promoted the recruitment of endogenous immune cells and reduced the expression of exhaustion markers on infiltrating T cells (146–148). Importantly, long-term follow-up studies have supported the clinical safety of IL-7/CCL19-secreting CAR-T cells (31, 149). Glioblastoma is a tumor with a complex TME (150). In addition to IL-7, IL-15, and CCL19 were used to generate CAR-T cells in a mouse glioblastoma model, yielding promising preclinical results (151). The findings show that 15 × 19 CAR-T cells exhibit improved proliferation, enhanced chemotactic ability, and more favorable phenotypic traits than conventional CAR-T cells *in vitro*. This improved performance is partly due to IL-15 and CCL19, which promote greater T cell infiltration into the tumor and strengthen resistance to exhaustion within the TME.

However, these homing strategies face the fundamental challenge of “chemokine and antigen heterogeneity.” Solid tumors rarely produce ligands uniformly; if sub-regions of the tumor mass fail to secrete the targeted chemokine, CAR-T cells may fail to infiltrate the entire lesion, leading to “tumor escape”. Additionally, high systemic levels of these chemokines in certain patients could “distract” engineered CAR-T cells, causing them to sequester in non-tumor tissues like the lungs or liver, thereby increasing the risk of off-target inflammation. Therefore, while chemokine receptor engineering improves infiltration, it must be integrated with strategies that address the biological complexity of the target landscape.

## 6 Biological constraints of target antigens: density and heterogeneity

The success of advanced CAR-T engineering is fundamentally predicated on the underlying biology of the target antigen, which remains one of the most significant constraints in solid tumor therapy. Unlike hematologic malignancies, in which antigens such as CD19 are often uniformly expressed across the malignant population, solid tumors exhibit extreme intra-tumoral heterogeneity (152). This biological diversity means that a single tumor mass may contain multiple subclones with varying antigen expression levels, leading to a “masking” effect in which antigen-negative or antigen-low cells escape the engineered immune response. Furthermore, selecting a truly tumor-specific antigen is exceptionally difficult, as most targetable proteins are also expressed at low levels in healthy tissues, posing a constant risk of on-target, off-tumor toxicity.

Mechanistically, the effectiveness of a CAR-T cell is governed by the antigen density threshold, the minimum number of antigen molecules per cell required to trigger a robust cytotoxic response. This density dependence is a critical factor in clinical failure; if the engineered CAR’s activation threshold is too high, tumor cells with low antigen density will survive. Conversely, if the threshold is too low, the CAR-T cells may attack healthy tissues that express trace amounts of the antigen. Modern engineering efforts, such as affinity tuning of the scFv or calibration of intracellular ITAM subunits, are designed specifically to “set” this threshold at a level that distinguishes between high-density tumor cells and low-density healthy cells (153). However, the inherent plasticity of solid tumors often leads to antigen escape, in which the tumor actively downregulates the target protein under the selective pressure of therapy, necessitating a shift toward more complex targeting strategies.

To address the challenge of antigen escape and heterogeneity, the field is increasingly moving toward logic-gated and combinatorial targeting. Dual-targeting CARs, which use “OR-gate” logic to recognize two distinct antigens simultaneously, ensure that the loss of a single target does not lead to immune evasion (154). Furthermore, “AND-gate” logic systems and microenvironment-sensing circuits, such as hypoxia-gated CARs, provide a safety mechanism by requiring both antigen presence and a specific environmental cue before activation. Beyond these cell-intrinsic designs, strategies that promote antigen spreading, such as oncolytic viruses, leverage the endogenous immune system to recognize a broader array of neoantigens released during tumor lysis. This multifaceted approach aims to bridge the gap between elegant synthetic engineering and the complex, heterogeneous reality of the solid tumor landscape, ensuring a more durable and comprehensive therapeutic outcome.

## 7 Combinatorial CAR-T engineering: synergistic potential and safety considerations

The integration of multiple CAR-T cell engineering strategies represents a promising avenue to overcome the multifaceted barriers of the tumor microenvironment; however, such combinatorial approaches introduce significant immunological complexity. Mechanistically, combining multiplex checkpoint gene disruption (e.g., PD-1, LAG-3, and TIGIT knockouts) with immunostimulatory interventions such as oncolytic viruses or nanomaterial-based cytokine delivery can synergistically enhance T cell activation, persistence, and tumor cytotoxicity. Oncolytic viruses promote tumor cell lysis and the release of neoantigens and danger-associated molecular patterns, thereby amplifying antigen presentation and local inflammation (155). When paired with checkpoint-deficient CAR-T cells that lack inhibitory feedback signaling, this can result in sustained and amplified effector responses (156).

However, the removal of multiple inhibitory pathways may disrupt immune homeostasis and increase the risk of uncontrolled immune activation. In such contexts, OV-induced inflammation, combined with hyperactivated CAR-T cells, could lead to excessive cytokine production, increasing the likelihood of severe CRS, ICANS, or autoimmune-like tissue damage. This concern is supported by clinical observations showing that enhanced immune activation through checkpoint blockade or combination immunotherapies can increase the incidence of immune-related adverse events (152, 157).

In addition to toxicity, immunogenicity represents another critical concern. Repeated exposure to viral vectors or synthetic nanomaterials may elicit host immune responses, including neutralizing antibodies or innate immune activation, which can limit CAR-T cell persistence and reduce therapeutic efficacy (158, 159).

Furthermore, gene-edited cells may exhibit altered immunogenic profiles, particularly in allogeneic settings where host-versus-graft responses may arise. To mitigate these risks, rational design of combinatorial CAR-T strategies should incorporate controllable elements, such as inducible safety switches (e.g., iCasp9), tunable activation systems, or transient expression platforms. Spatial and temporal regulation of immune activation, for example, restricting OV replication to tumor tissue or using locally activated nanomaterials, may further reduce systemic toxicity. Importantly, previous studies combining oncolytic viruses with immune checkpoint blockade have demonstrated enhanced antitumor efficacy but also increased immune-related toxicities, underscoring the need for careful dose optimization and safety control (156).

## 8 Challenges in clinical translation and implementation

The rapid expansion of CAR-T engineering approaches has generated a diverse set of strategies with distinct mechanisms, advantages, and translational challenges. A comparative evaluation of these platforms highlights important differences in their clinical readiness and feasibility.

From a mechanistic perspective, current strategies can be broadly grouped into:

- genetic engineering approaches (e.g., CRISPR/Cas-mediated editing, shRNA knockdown),
- synthetic circuit-based designs (e.g., logic-gated CARs, switchable systems), and
- microenvironment-modulating platforms (e.g., nanomaterials and oncolytic viruses).

These categories differ substantially in their maturity and translational trajectory. Genetic modifications such as single-gene knockouts (e.g., PD-1 disruption) and cytokine-armed CAR-T cells have already entered early-phase clinical trials, reflecting relatively greater translational readiness. In contrast, more complex strategies such as multiplex gene editing, multi-input logic circuits, and nanomaterial-integrated CAR-T systems remain largely at the proof-of-concept stage due to increased manufacturing complexity and regulatory challenges.

Each approach presents distinct advantages and liabilities. Gene editing enables durable functional reprogramming but raises concerns regarding off-target effects and genotoxicity (129). Synthetic circuit designs offer enhanced specificity and control but require sophisticated engineering and validation. Nanomaterials and oncolytic viruses provide powerful tools for modulating the tumor microenvironment; however, their integration introduces additional considerations regarding immunogenicity, pharmacokinetics, and combination product regulation (98).

Importantly, translational feasibility is determined not only by biological efficacy but also by manufacturability, scalability, and cost-effectiveness. Simpler and modular designs are more likely to achieve near-term clinical translation, whereas highly complex, multi-component systems may face significant barriers despite promising preclinical performance. **Table 2** outlines the developmental stages of each strategy despite these clinical-translational barriers.

**Table 2:** Developmental landscape of CAR-T cell engineering strategies.

Strategy	Mechanism	Development stage	Representative examples	Translational notes
Affinity-tuned CARs	Density-dependent antigen recognition	Preclinical to early clinical	Low-affinity HER2 CARs	Promising for safety; requires precise calibration
Logic-gated CARs (AND/NOT switches)	Multi-antigen recognition control	Preclinical to early clinical	SynNotch, dual CAR systems	High specificity; increased design complexity
Switchable/adaptor CAR systems	External control of CAR activation	Early clinical (limited)	Small molecule-regulated CAR	Flexible control; regulatory complexity CARs
Hypoxia-/tumor-gated CARs	Microenvironment-restricted activation	Preclinical	Hypoxia-inducible CARs	Improves specificity; early-staged development
Oncolytic viruses (in combination)	Tumor lysis + immune activation	Early clinical/clinical adjunct	OV + CAR-T Combinations	Strong synergy; immunogenicity and dosing need optimization
Nanomaterial-assisted CAR-T	Local delivery, TME modulation	Preclinical (*in vivo*	Liposomes, nanozymes, CAR-T biohybrids	Strong preclinical efficacy; limited clinical translation so far
Injectable biomaterial platforms(hydrogels)	Localized CAR-T delivery/support	Preclinical	CAR-T + hydrogel systems	Promising for solid tumors; scalability unclear
Single-gene editing (e.g., PD-1 KO)	Removal of inhibitory signaling	Early clinical (first-in-human ongoing)	PD-1-deficient CAR-T cells	Relatively mature; manageable manufacturing complexity
Multiplex gene editing	Simultaneous targeting of multiple pathways	Preclinical (*in vivo*)	PD-1/LAG-3/TIGIT KOCAR-T	High efficacy potential; major manufacturing/regulatory barriers
Cytokine-armored CAR-1	Autocrine cytokine support (e.g., IL-12, IL-15)	Early clinical	IL-12 or IL-15 secreting CAR-T	Potent but requires tight toxicity control

## 9 Clinical translation of next-generation CAR-T cell engineering strategies

While most advanced CAR-T engineering strategies remain in preclinical development, several have entered early-phase clinical trials, offering important insights into their translational feasibility. Among these, CRISPR/Cas9-mediated gene editing has emerged as a clinically viable approach. A first-in-human phase I trial (NCT03399448) demonstrated the feasibility of multiplex CRISPR-edited T cells targeting PD-1, TRAC, and TRBC loci, with no unexpected toxicities and evidence of prolonged persistence in patients with advanced malignancies (160). Similarly, targeted disruption of PD-1 in CAR-T cells has been evaluated in early clinical studies. For example, a phase I trial (NCT03525782) investigating PD-1 knockout MUC1-targeted CAR-T cells in patients with advanced solid tumors reported a favorable safety profile, with no severe cytokine release syndrome observed and preliminary signals of disease stabilization. In addition to autologous approaches, allogeneic gene-edited CAR-T therapies are rapidly advancing. The CRISPR-edited anti-CD19 CAR-T product CTX110 (NCT04035434) demonstrated encouraging clinical activity in relapsed/refractory B-cell malignancies, with reported overall response rates exceeding 50% in early analyses, supporting the feasibility of scalable “off-the-shelf” CAR-T platforms (161). Similarly, the PD-1 knockout allogeneic CAR-T therapy CB-010, evaluated in the ANTLER trial, has shown promising response rates and manageable safety profiles in patients with non-Hodgkin lymphoma (162). Beyond gene editing, safety switch systems have also demonstrated clinical utility. The iCasp9 suicide switch has been validated in multiple clinical settings, enabling rapid pharmacological elimination of CAR-T cells in cases of severe toxicity, thereby significantly improving safety control. After rimiducid administration, peripheral blood CAR-T cell counts dropped sharply, and this lowered level persisted following a subsequent dose. At six weeks, the CAR-T cells began to expand again but remained responsive to control by rimiducid (163, 164). Furthermore, switchable CAR-T platforms, including adaptor-mediated systems that enable reversible, dose-dependent activation, are currently in early clinical investigation. These systems provide an additional layer of control over CAR-T cell activity and may reduce on-target/off-tumor toxicity (164).

Despite these advances, current clinical data remain limited to early-phase trials with relatively small patient cohorts. Challenges such as heterogeneous responses, limited persistence in solid tumors, antigen escape, and potential cumulative toxicity in multiplex-engineered systems remain to be fully addressed. Therefore, although next-generation CAR-T strategies demonstrate clear translational promise, larger and more comprehensive clinical studies are required to validate their long-term efficacy and safety.

## 10 Conclusion and future perspective

CAR-T cell therapy has demonstrated excellent efficacy in treating hematologic malignancies, and CAR-T-based personalized immunotherapy has led to notable remissions. However, this mode of immunotherapy is still in its early stages, and specific challenges persist. For example, the CAR-T-associated CRS and neurotoxicity are significant side effects. Additionally, noteworthy factors include the limited persistence of CAR-T cells, their failure to home to tumor sites, inhibitory factors within the TME, exhaustion induced by metabolic stress, and inhibitory signals. Addressing these barriers will move the field one step closer to translating CAR-T cell therapy into a durable and widely applicable cancer treatment.

Recent advances reviewed here highlight a shift toward rational, multi-layered engineering strategies. Self-regulating CAR designs and switch-on/switch-off CAR systems provide critical safety controls to mitigate CRS and neurotoxicity. Other strategies, such as integrating nanomaterials and OVs, offer powerful means to remodel the TME, enhance immune infiltration, and sustain CAR-T cell function under hostile metabolic and immunosuppressive conditions. At the cellular level, precision genome engineering using CRISPR-based editing and multiplexed shRNA has enabled targeted rewiring of inhibitory signaling pathways, exhaustion programs, and stress-response networks, thereby improving persistence and antitumor efficacy. Complementing these efforts, chemokine-based strategies realign CAR-T cell migration with tumor-specific cues, overcoming trafficking and infiltration deficits that have long limited efficacy in solid tumors. Although a long journey remains, these interdisciplinary innovations are paving the way for next-generation CAR-T products that combine robust antitumor activity with built-in safety mechanisms and improved durability.

While next-generation CAR-T cell engineering strategies offer substantial therapeutic promise, their increasing biological sophistication introduces significant challenges in manufacturing, scalability, and cost. Advanced approaches such as multiplex genome editing, synthetic logic-gated circuits, and nanomaterial-based modifications require additional processing steps, more complex quality control pipelines, and stringent validation procedures, all of which complicate production under Good Manufacturing Practice (GMP) conditions (165). One major concern is the impact of these modifications on batch-to-batch consistency. Variability in gene editing efficiency, vector integration, and transgene expression can lead to heterogeneity in CAR-T cell products, potentially affecting both safety and efficacy. For instance, multiplex CRISPR editing increases the risk of variable editing outcomes and off-target effects, necessitating extensive release testing and characterization. Similarly, incorporating synthetic circuits or nanomaterials introduces additional sources of variability that are difficult to standardize across large-scale production (166, 167).

To address these limitations, several strategies are being actively explored. Automated, closed-system manufacturing platforms have been developed to improve reproducibility and reduce operator-dependent variability. In parallel, the development of allogeneic “off-the-shelf” CAR-T cells, enabled by gene editing to eliminate graft-versus-host disease and host rejection, offers a promising route to scalable and cost-effective production. Despite these advances, achieving a balance between engineering complexity and manufacturing feasibility remains a critical challenge, and the field’s future success will be measured by its ability to deliver these sophisticated therapies consistently and affordably to a broad patient population.

## Glossary

ADRB2: Beta-2 adrenergic receptor
B-ALL: B cell acute lymphoblastic leukemia
CAR: Chimeric antigen receptor
CCRs: Chimeric co-stimulatory receptors
CRS: Cytokine release syndrome
CT-INPs: CAR-T cell-INP biohybrids
CTLA4: Cytotoxic T-lymphocyte associated antigen 4
Cu: Copper
DCs: Dendritic cells
DSB: Double-strand break
ECM: Extracellular matrix
EGFR: Epidermal growth factor receptor
ERT2: Estrogen receptor ligand-binding domain T2
FDA: U.S. Food and Drug Administration
GCV: Ganciclovir
GLUT-1: Glucose transporter 1
HA: Hyaluronic acid
HD/TMD: Hinge and transmembrane domain
HDAC11: Histone deacetylase 11
HIF1α: Hypoxia-inducible factor 1-alpha
HREs: Hypoxia-responsive elements
HSV-TK: Herpes simplex virus thymidine kinase
huCART19: Humanized CD19 chimeric antigen receptor T-cell product
HyaII: Hyaluronidase
ICANS: Immune effector cell-associated neurotoxicity syndrome
iCasp9: Inducible caspase 9
ICD: Intracellular co-stimulatory domains
IFNs: interferons
IFN-γ: Interferon gamma
IL: Interleukins
IL-10: Interleukin-10
INPs: Indocyanine green nanoparticles
ITAMs: Immunoreceptor Tyrosine-based activation motifs
LAG3: Lymphocyte activation gene 3 protein
LiCAR: Light-inducible CAR
LNPs: Lipid nanoparticles
LOV2: Light-oxygen-voltage 2
MDSCs: Myeloid-derived suppressor cells
MHC: Major histocompatibility complex
NLS: Nuclear localization sequence
NSCLC: Non-small cell lung cancer
ODD: Oxygen-dependent degradation
OVs: Oncolytic viruses
PDAC: Pancreatic ductal adenocarcinoma
PDCD1: Programmed cell death protein 1
PD-L1: Programmed cell death ligand 1
PGC1-α: Peroxisome proliferator-activated receptor gamma coactivator 1α
PI3K: Phosphoinositide 3-kinase
PRDM1: PR domain zinc finger protein 1
PTT: Photothermal therapy
scFv: Single-chain variable fragment
shRNA: Short-hairpin RNA
SspB: Stringent starvation protein B
SsrA: Small stable RNA A
TAA: Tumor-associated antigen
TAMs: Tumor-associated macrophages
TCF1: T cell factor 1
TCM: Central memory T cell
TCR: T cell receptor
TGFBR2: TGF-β
TGF-β: Transforming growth factor-beta
TIGIT: T-cell immunoreceptor with immunoglobulin and immunoreceptor tyrosine-based inhibitory motif domains
TIM3: T cell immunoglobulin mucin domain-containing protein 3
TKOs: Triple knockouts
TME: Tumor microenvironment
TNF-α: Tumor necrosis factor-alpha
TPEX: Precursor exhausted T cell
TRAF2: TNF receptor-associated factor 2
Tregs: Regulatory T cells
TRUCKs: T cells redirected for universal cytokine-mediated killing
VSV: Vesicular stomatitis virus
α-GalCer: α-galactosylceramide.
